# Intragenic deletions from whole genome sequencing of 1054 suicide deaths

**DOI:** 10.21203/rs.3.rs-9942744/v1

**Published:** 2026-07-27

**Authors:** Hilary Coon, Emily DiBlasi, Thomas Nicholas, Eric Monson, Elliott Ferris, Andrey Shabalin, Logan Yefimov, Brooks Keeshin, Amanda Bakian, Seonggyun Han, Lisa Baird, William Callor, Michael Staley, Dierdre Amaro, Qingqin Li, Virginia Willour

**Affiliations:** University of Utah; University of Utah; University of Utah School of Medicine; University of Utah School of Medicine; University of Utah School of Medicine; University of Utah School of Medicine; Utah Department of Health; CHDI Management, Inc; University of Iowa

## Abstract

Suicide is an urgent public health crisis that claimed over 49,000 lives in the US in 2023. While genome-wide association studies of suicide are beginning to reveal genetic risk attributable to common variants with small effects on liability, these results explain only a fraction of the substantial proportion of risk due to genetics known to contribute to suicide mortality. As with other complex health conditions, some of this unexplained genetic risk is likely due to rarer variants with larger effects on liability. Using whole genome sequencing data from 1,054 population-ascertained suicide deaths from the Utah Suicide Mortality Research Study (USMRS) jointly processed with 1,230 controls, we investigated intragenic deletions as a class of genomic variation likely to disrupt gene function. To minimize false positives, deletions were limited to those found in large publicly available control datasets (1000 Genomes, GnomAD, and Centers for Common Disease Genomics) and where replication of deletions occurred across two cohorts within the USMRS suicides. Deletions meeting these filters were manually validated. Eleven deletions had at least 2-fold increase in frequency in suicide deaths vs. controls (range 2.28 to 4.46). Implicated genes were associated with mental health conditions (*MPST, IL4R, CDH13*), epilepsy (*CLCA4*), intellectual disability (*ZNF44*), neuronal function (*OSBPL2*), metabolic function (*FBOX36*), lipid metabolism (*TM9SF3*), immune functions (*PIPOX, IL4R*), and Alzheimer’s disease (*ZHX3, LMNTD1*). Pending replication, these results may help prioritize biological pathways for future functional studies with the goal of increasing our understanding of risk mechanisms leading to suicide mortality.

## Introduction

In the U.S., the suicide death rate is the highest it has been in over 80 years;^1^ suicide prediction and prevention are top public health priorities. Identification of risk factors leading to suicide death remains challenging due to the complexity of suicide risks; however, genetic factors comprise one important aspect of risk that may allow substantial progress in bridging this knowledge gap, as estimates of heritability are ~ 50%.^2–4^

Recent genome-wide association study (GWAS) results based on large cohorts have begun to reveal common genetic factors associated with suicide outcomes.^5–10^ While these findings represent significant progress, due to availability of data they are largely dominated by cohorts of living individuals with suicidal ideation (SI) or suicide attempt (SA), not suicide mortality. The resulting focus on the close association between underlying polygenic risk of psychiatric disorders with polygenic risks of suicide outcomes in these studies may reflect the demonstrated strong clinical associations of psychopathology with SI and SA.^11^ However, recent data suggests that the risk of suicide mortality, as compared to risk of SI/SA, may also have strong non-psychiatric risk associations,^12–15^ highlighting a knowledge gap that could be bridged through closer study of genetic variation among suicide deaths.

In addition, the recent GWAS studies of suicide outcomes provide estimates of SNP heritability ranging from 3.9–5.7%, considerably less than the heritability estimates of suicide outcomes of 30–50% from the classical behavioral genetic designs involving aggregated family and twin studies^3, 16–19^. This “missing heritability” likely derives from effects of rare genomic variation and/or from the contribution of gene x gene or gene x environment effects.^20–21^ A recent exome-wide study of rare potential functional single nucleotide variants in Veterans^22^ provides an important step to begin to bridge this knowledge gap, though that study focuses on the outcomes of suicidal thoughts and behaviors rather than suicide mortality.

These knowledge gaps regarding specific risks associated with mortality and limited knowledge of contributions of rare variation can be addressed by investigating large samples of population-ascertained suicide deaths regarding changes in rarer genetic variation, particularly structural variation.^23^ Structural variants (SVs) are a broad class of genome variation that is extremely diverse in type and size (typically 50–100 base pairs to several megabases).^24^ SVs are present in all human genomes and often have severe functional consequences due to their ability to disrupt genes, cause gene fusions, rearrange regulatory elements and alter gene dosage.^25^ SVs have historically been harder to study than common variation data due to limitations associated with the necessary sequencing technologies and detection algorithms.^24^ Thus, like rarer SNVs, structural variants have largely been excluded from large-scale genetic studies even though rare, functional genetic variation may play a crucial role in understanding the complexities of suicide risk.^26^

The current study provides results from a comprehensive study of whole genome sequence (WGS) data from a large sample (N = 1,054) selected from a population-ascertained suicide mortality study.^27^ This study design addresses previous challenges faced by studies of rare variation in suicide mortality, including small cohorts, reliance on genotyping rather than WGS data, and/or ascertainment within individuals with specific clinical diagnoses. Using WGS data, emerging approaches can be used to more reliably detect and call SVs.^24^ This genome-wide investigation focuses on deletions overlapping coding sequence of genes; these represent genomic events most likely to affect gene function. We have chosen to focus on deletions as they are more confidently detected by available robust computational tools.^24,28^

## Methods

**Figure 1:**
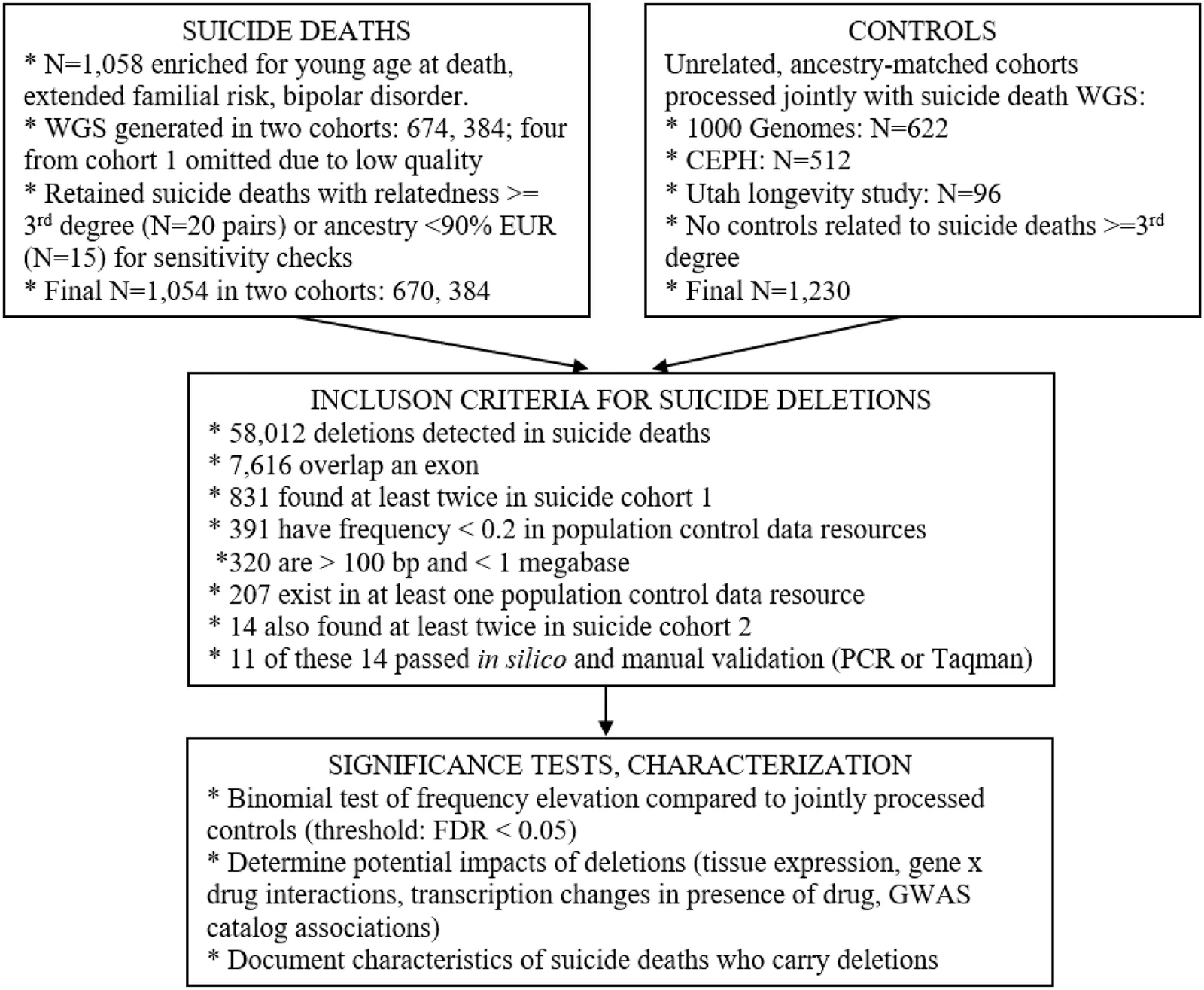
Cohort descriptions and filtering of deletions.

An overview of the study cohorts, filtering steps, and testing for deletion significance is shown in Fig. 1.

Data resources from suicide deaths. Data from Utah suicide deaths used in this study were available through a long-term collaboration with the Utah State Office of the Medical Examiner (OME). Suicide determination was made by the OME following detailed investigation of the scene and circumstances of death. High quality DNA was extracted from whole blood obtained by the OME as a part of routine autopsies as previously described.^27^ Linking identifiers from suicide deaths were securely transferred by the OME to staff at the Utah Population Database (UPDB; https://uofuhealth.utah.edu/huntsman/utah-population-database). The UPDB is a statewide database with > 27 million data records, including demographics, electronic health records data, and genealogical records. UPDB staff unrelated to the study linked OME data from suicide deaths to UPDB health data, and then secure data transfer methods were used to provide a limited use dataset to the research team. This study was approved by Institutional Review Boards at the University of Utah, Intermountain Healthcare, and the Utah Department of Health and Human Services.

Selection of the initial 1,058 suicide deaths for generation of whole genome sequencing (WGS) data was done based on factors associated with increased likelihood of greater genetic risk, including younger age at death, evidence for significant extended familial risk of suicide death using Utah genealogical data,^29^ and diagnoses of bipolar disorder, which is associated with high risk of suicide death.^30^

Control data. Joint processing of WGS data from Utah suicide deaths was done with individual WGS data from 1,230 controls unrelated to each other or to the suicides, and matching the predominantly Northern European ancestry of the Utah population. These unrelated, ancestry-matched controls were from three sources: 1) 622 from the 1000 Genomes Project (1000Genomes);^31^ 2) 512 from the Centre d’Etude du Polymorphisme Humain (CEPH) genetic reference families;^32^ and 3) 96 from a Utah study of longevity.^33^

Relatedness and ancestry. Pairwise relatedness was computed using genetic identity by descent in PLINK.^34^ Among suicide deaths, 20 pairs with third-degree relatedness or closer were retained to allow for correction of initial results and prioritization for potential future analyses if familial transmitted variants were observed. Prior to selection for this study, all controls related to suicide deaths at the level of third degree or closer were eliminated.

However, all suicide deaths were retained regardless of ancestry. Sensitivity tests were implemented to determine the extent to which any result was driven by non-European carriers of the deletions. Ancestry was determined through comparison of genotypes to reference population data available in the 1000Genomes using the kgp2anc algorithm (https://github.com/freeseek/kgp2anc). Overall, data from Utah suicides with WGS revealed predominantly European ancestry (98.10%); only 15 suicide deaths had < 90% EUR ancestry.

Whole genome sequencing pipeline. WGS data were generated on the 1,058 Utah suicide deaths and processed jointly with data from 1,230 controls. All data were generated using Illumina NGS technology with an average read depth of ~ 30x. Alignment, variant calling, and joint genotyping of suicide deaths and control WGS datasets was performed at the Utah Center for Genetic Discovery (UCGD) Core Facility, part of the Health Sciences Center Cores at University of Utah. The UCGD pipeline (v 2.13.77) called variants using the Sentieon software package,^36^ which incorporates GATK best practices^37–38^ with other innovative elements. Use of this UCGD pipeline has been described in detail elsewhere.^39^ Briefly, sequence reads were aligned to GRCh38 (Genome Reference Consortium Human Build 38) using BWA-MEM.^40^ The Haplotyper algorithm in Sentieon^36^ was used to produce genomic Variant Call Format (gVCF) files.

Final suicide death WGS cohorts. Suicide deaths were processed in two cohorts (N = 674 and N = 384); data from four in the first cohort were omitted due to low quality, leaving N = 670 and N = 384, total N = 1,054. The gVCF files were then combined and jointly processed with data from the 1,230 individual controls. Table 1 presents descriptive data from the two suicide death cohorts, showing more individuals with older age at death and more females in cohort 2 compared to cohort 1 (age test: t = 10.93, p < 0.0001; sex test: chi-square = 11.75, p = 0.0006).

Structural variant (SV) calling. Detection of structural variants (SVs) was done using Smoove (version 0.2.6l; https://github.com/brentp/smoove) and Manta (version 1.6.0),^41^ again using the GRCh38 reference genome. Smoove and Manta produce VCF files that contain deletions and duplications, and unclassified breakends; Smoove additionally includes inversions and Manta includes insertions. Genic information (Ensembl GRCh38.p12) and Duphold^42^ quality metrics were added to the VCF files using the Smoove annotate and duphold commands. SVAFotate^43^ was also used to annotate population frequencies of deletions from public WGS control datasets including the Genome Aggregation Database (GnomAD),^44^ the Centers for Common Disease Genomics (CCDG),^45^ the full 1000Genomes cohort,^31^ and the full CEPH cohort.^32^ The reciprocal overlap fraction requirement of 0.8 was used to match SV calls between the suicide cohort and these public datasets in SVAFotate.

Filtering criteria for deletions. Filtering was performed using Slivar,^46^ and filtered to minimize artifacts and maximize potential functional deletions using these criteria: 1) overlap an exon using Smoove gene annotations; 2) pass Duphold^42^ quality metrics (Duphold_pass flag and fold change DHFFC < 0.7) and be present in at least suicides in suicide cohort 1; 3) have frequency < 0.2 in at least one of the large control WGS data resources noted above to remove more common deletions; 4) be > 100 base pairs (bp) and < 1 megabase (Mb); 5) exist at least once in the large control WGS resources to remove likely artifacts; and 6) be present also in at least two suicides in cohort 2 (N = 384) to provide internal validation.

Statistical tests. For deletions meeting these criteria, frequencies in suicide deaths were compared to frequencies in the jointly processed control sequence data using exact binomial tests. Deletions with significant fold increase in frequency over jointly called control data at a false discovery rate < 0.05 were retained for manual validation. Frequencies of these deletions were checked in the GnomAD non-Finnish European “non-neuro” subset (GnomAD v2.1, N = 16,684), which omits data from individuals with neuropsychiatric and neurological conditions.^47^ Because these GnomAD data were not jointly called with the suicide data, these aggregated control frequencies were checked for comparison only.

Manual validation of deletions. Smaller deletions (less than 5,000 bp) were verified by PCR and subsequent electrophoresis of the PCR product on agarose gels. First, primers flanking the putative deletions were designed using the Primer3 program (v. 0.4.0). 50 ng of template DNA was then amplified with these primers in a PCR reaction using Q5^®^High-Fidelity DNA Polymerase (New England Biolabs^®^) per manufacturer’s recommended conditions. PCR product was subsequently electrophoresed on 1XTBE 2% agarose gels in the presence of Ethidium Bromide and then visualized on a Fotodyne^™^ FOTO/Analyst^™^ Workstation. The deletion was verified when product bands of two different sizes were present after PCR, one band being the larger wild type allele and the other band being a smaller deleted allele. Larger deletions (greater than 5,000 bp) were verified using pre-designed Taqman^™^ Copy Number Assays purchased from Thermo Fisher Scientific. Quadruplicate reactions were carried out per manufacturer’s recommendations, and the data was analyzed with CopyCaller^®^ Software.

Additional validation was done using SAMPLOT^48^ to visualize the deletions *in silico* for comparison with manual validation results and to obtain indications of read depth sequence alignments and confidence intervals of calls in the region of the deletion. Specifically, each deletion was inspected in the called region for losses in the depth of coverage or split or discordant read signatures.

Characterization of implicated genes and of SNPs within the deletions. Potential functional impact of SNPs within each significant deletion was characterized using public data from the GTEx consortium (https://gtexportal.org/). Gene-drug interactions were compiled using the DGIdb resource^49^ and gene expression level changes in the presence of drugs were compiled using DRUGBANK.^50^ Additional characterizations of the genes implicated by the deletions included publicly available genetic associations from the GWAS Catalog.^51^

Clinical data. Once SVs were identified and validated, additional electronic health records (EHR) data linked to suicide deaths were then used to provide descriptive information regarding possible variation in the proportion of decedents with co-occurring health conditions by deletion in order to direct possible future studies. Diagnostic data from the International Classification of Diseases (ICD-9 and ICD-10; https://archive.cdc.gov/www_cdc_gov/nchs/icd/) system in the EHR was tabulated for suicides with deletions. Diagnoses were collapsed into phecodes,^52^ curated groups of ICD codes created to capture clinically meaningful phenotypes, using the hierarchical classification of diagnoses in the PheWAS catalog (PheCode Map X).^53^ Diagnoses that occurred within one week prior to death were excluded to eliminate diagnoses associated with the final fatal suicide event rather than those reflecting prior risk. Phecodes relating to mood disorders, bipolar disorders, substance use disorders, psychotic disorders, anxiety disorders, sleep disorders, and disorders associated with pain were compiled (see Supplemental information for specific ICD codes within each phecode).

## Results

Sequencing quality control. Overall, WGS data in the Utah suicide deaths was of high quality, with coverage from 25x to 48x across both batches, averaging 30x. In addition to the four cases dropped from suicide cohort 1 for poor quality noted above, data from nine cases in this cohort with lower alignment (one at 77.6%, four at 80%−90%, four at 90%−95%), and six cases from the second suicide cohort with lower alignment (one at 74.2%, two at 80%−90%, and three at 90%−95%) were retained for analysis and scrutinized at the manual validation stages. Descriptive demographic data was available for the subset of suicide deaths with available verified health records data (N = 1,049).

SV discovery and filtering. Figure 1 summarizes the deletion filtering. In the initial cohort of 670 suicide deaths, used as the discovery set, 58,012 deletions were discovered. While additional structural variants were apparent (e.g., insertions, inversions), this study focused on deletions as those that are the most robustly detected.^24,28^ Of the 58,012 deletions, 7,616 crossed an exon using the Smoove_gene annotation. At the next step, 831 deletions passed Duphold quality parameters and were present in at least two suicide deaths in cohort 1. Using the SVAFotate tool, each deletion was compared to the public data cohorts (GnomAD, CCDG, 1000Genomes, and CEPH). Deletions were deemed to be present in controls if there was at least 0.8 reciprocal overlap in deletion boundaries. We only retained those with a frequency of < 0.2 in at least one of these control cohorts to eliminate common events, resulting in 391 retained deletions. Of these 391, 29 were >1Mb, and 42 were < 100bp; these were omitted as likely artifacts.

After these filtering steps, there were 320 deletions, 113 of which were not found in any of the large external control cohorts. Most of these previously undescribed deletions (N = 96, 85%) were with imprecise boundaries in the suicide cohort, suggesting these deletions may represent spurious findings; these deletions will require future detailed studies. For this study, we retained only the 207 deletions that were also found in at least one of the large external control datasets. Our next step was to determine the presence of these 207 deletions to the second cohort of 384 suicide deaths. We required precise base-pair matching of the start and stop of the deletion in two suicides in the second cohort of 384 suicides for this replication step. Of the 25 deletions that met this requirement, there were 14 deletions with at least two occurrences in this second cohort, and with at least a two-fold increase in frequency of each deletion over and above its frequency in jointly processed controls.

Manual validation and statistical testing of the 14 deletions. Validation for seven of the smaller deletions was done using PCR (*PIPOX, TM9SF3, ZNF44, OSBPL2, ZHX3, APOOL, MPST*; see Supplemental Tables S1-S2 for primers and details). Primers for the *LILRA1* deletion (chr19:54601316–54601631) were not possible to design given extensive homology across the region of the deletion with three other regions on chromosome 19. For the *APOOL* deletion (chrX:85087992–85088221), only seven of 16 suicides clearly showed the deletion. These two deletions were therefore not studied further in this analysis. Validation of *MPST* was also carried out with a Taqman assay because the PCR validation only amplified the smaller, deleted allele. This Taqman assay showed evidence of varying copy numbers of this SV in both suicides and controls. Though results still implied case-control differences, this variation suggests interpretation of this deletion should be approached with caution. For the other deletions validated by PCR, all suicides determined to have the deletion by *in silico* methods (SAMPLOT)^48^ also showed validation with PCR except for data from one suicide for the *TM9SF3* deletion, leaving 10 suicides with that deletion (case frequency = 0.00476, fold increase over controls = 3.33). For the larger deletions requiring Taqman validation, *FBXO36, LMNTD1, CLCA4, FBX036*, and *IL4R* were manually validated for all suicides with these deletions. For *CDH13*, two of the 20 suicides initially identified as having the deletion failed to validate, leaving 18 with the deletion (case frequency = 0.000857, fold increase over controls = 2.06).

Figures representing successful *in silico* and manual validations for 11 remaining deletions can be found in the supplemental materials. Table 2 gives details of each validated deletion. These 11 deletions were significantly elevated over the frequency in jointly processed WGS data from controls at a false discovery rate (FDR) of 0.05.

SNP-level and gene-level associations of significant deletions. For the 11 significant deletions, tissue associations are summarized in Table 3 for SNPs within each significant deletion with overlapping in GTEx data. Brain expression of SNPs within deletions was found for *FBXO36*, *ZNF44*, *CLCA4*, and *IL4R*. Further details of the GTEx SNP-level data is given in Supplemental Table S3. Deletions in *MPST, LMNTD1, IL4R*, and *CDH13* implicated gene-drug interactions from data in the Drug-Gene Interaction Database (DGIdb).^49^ Table 3 lists additional gene transcriptomic changes in the presence of drugs (DRUGBANK)^50^ and also gives a summary of associations for each gene found in the GWAS catalog.^51^ Further discussion of the associations with these resulting genes is provided below.

Phenotypic associations. There were no related pairs among the suicide deaths sharing validated deletions in Table 2. None of the validated deletions occurred in individuals with < 95% European ancestry with the exception of the deletion in *TM9SF3* on chromosome 10. This deletion was carried in two suicides with < 95% European ancestry, one with 84.1% European ancestry, 15.0% Admixed American ancestry, and 0.9% South Asian ancestry, and the other with 81.3% European ancestry, 17.9% East Asian ancestry and 0.8% Admixed American ancestry. Removing these two occurrences from the data lowered the observed frequency to 0.00381, leaving a fold increase of 2.67 and a binomial test significance of 1.19E-02 which remained significant at FDR (0.05).

Supplemental Table S4 presents descriptive aggregated demographic and clinical data from suicides carrying each deletion and also the overall frequencies in all 1,049 suicide deaths with WGS data and verified health records data, and the subset of 1,030 with sufficient ICD data to compute phecodes (two suicides with deletions, one with *IL4R* and one with *FBX036*, and 17 without any deletions had insufficient ICD data to compute phecodes). The variability in demographic and clinical data across those carrying deletions is presented to provide descriptive information, but not subjected to statistical tests as numbers of individuals with each deletion are low.

## Discussion

This study represents an extensive investigation of structural variation, specifically intragenic deletions, in a large cohort of suicide deaths. Our analysis of whole genome sequencing (WGS) data from 1,054 suicide cases and 1,230 controls identified 11 internally replicated, manually validated deletions with significant enrichment in suicide deaths compared to controls. The use of WGS data allowed for a comprehensive analysis of deletions, overcoming limitations of our previous studies that relied on genotyping arrays which do not contain the detailed sequence information.^54–55^ The deletions found in this study impact genes with associations that offer insights into potential genetic mechanisms leading to suicide mortality risk. These results builds upon evidence from previous studies of common single nucleotide polymorphisms (SNPs) and studies of rare single nucleotide variants (SNVs).

Unsurprisingly, several of the genes with validated deletions have been associated with a range of mental health conditions. *MPST*, which is involved in cellular energy metabolism, has been associated with stress-induced depressive sypmptoms^56^ and schizophrenia.^57^ *IL4R*, a gene that modulates immune and inflammatory responses, has also been implicated as a modulator of stress exposures resulting in later depression, anxiety, and/or cognitive impairment.^58–59^ *CDH13*, which regulates synapse formation and neuronal migration has been associated with several neuropsychiatric conditions, including depression, schizophrenia, bipolar disorder, and ADHD.^60–61^

In addition to psychiatric risks, genes in this study show associations with neurological conditions, neurodevelopment, and neurodegeneration. *CLCA4*, which is a chloride channel regulator, has been linked to epilepsy risk.^62^ *ZNF44*, a transcription factor, has been associated with intellectual disabilities,^63^ highlighting a potential overlap between suicide risk and neurodevelopmental conditions that has been previously documented in the Utah suicide data resource.^64^ Both *ZHX3*, a stress-related RNA regulator, and *LMNTD1*, a regulator of cell proliferation, have been implicated in Alzheimer’s disease.^65–66^ In addition, though not directly implicated in neurodegeneration, *OSBPL2*, a lipid transporter, has been associated with cholinergic neuronal differentiation;^67^ degeneration of cholinergic neurons is a common phenomenon in Alzheimer’s disease.^68^ *PIPOX* has functions relating to neuroprotection, cellular stress, and aging processes.^69^

Notably, several of the genes associated with deletions in our study, including *PIPOX*, *ZHX3, CDH13*, and *LMNTD1* have also been implicated in previous GWAS related to psychiatric, neurodevelopmental, and neurodegenerative disorders.^70–75^ In addition to gene-level associations, SNPs within the implicated deletion regions in this study also revealed potential relevance for psychiatric or neurological risk. In particular, GTEX SNP-specific results revealed substantial brain expression for *CLCA4* and *IL4R*.

Gene-drug interactions and drug-related transcriptomics also suggest potential interesting associations. Of particular interest for suicide outcomes, a gene-drug interaction has been noted between *CDH13* and deutetrabenazine which is indicated for tardive dyskinesia and chorea in Huntington’s disease, but has an adverse event profile that includes depression, insomnia, and suicidality.^76–77^ Upregulation of *CHD13* with folic acid provides an interesting connection with a recent study showing an association between several studies linking suicide outcomes to folic acid.^78–82^ *CDH13* additionally shows gene-drug interactions with other drugs used to treat neurological disorders (tetrabenazine) or psychiatric disorders (reserpine, perphenazine, dextroamphetamine sulphate),^49^ and with compounds used in neurological or psychiatric research (tritiated dihydrotetrabenazine, iodovinyltetrabenazine, [125I]7-Azido-8-iodoketanserin, Carbon-11 labeled dihydrotetrabenazine).^49^ The upregulation of *ZHX3* with disulfiram, an aldehyde dehydrogenase inhibitor used for alcohol addiction corroborates evidence from the GWAS catalog suggesting the possible importance of this gene in alcohol-related disorders that have been notably implicated in suicide risk.^83^ Transcriptional associations with valproic acid, a histone deacetylase inhibitor associated with psychiatric disorders, seizures, and migraine, was found across several of the implicated genes in our study (*PIPOX, TM9SF3, ZNF44, OSBPL2, ZHX3*). Valproic acid is known to alter expression of many genes involved in neuronal function.^84^

The genes implicated in this study also suggest the potential importance of non-psychiatric aspects of health in suicide risk. This result is possibly unsurprising given recent work implicating health risks beyond neuropsychiatric associations with suicide mortality.^12–15,^ In particular, the deletions in *PIPOX* and *IL4R* specifically suggest involvement of immune function and inflammation,^87–91^ which have previously been associated with suicide outcomes.^92–95^ In our study resource, the importance of these processes has been implicated a via associations with pain^85–86^ and with environmental exposures.^96–97^ Of note, gene-drug interactions and transcriptomic drug-gene changes also highlight immune function across several of the genes implicated in this study. Notably, regulatory changes were seen with cyclosporine for *PIPOX, TM9SF3, ZNF44, OSBPL2, ZHX3*, and *IL4R*. Cyclosporine is an immunosuppressant, which is known to alter expression of genes across a broad network of immune pathways.^98^ Other risk processes suggested by our findings include metabolic function (*FBXO36*, F-box protein 36, a ubiquitin protein)^99^ and lipid metabolism (*TM9SF3*, transmembrane 9 superfamily member 3, a regulator of Golgi homeostasis).^100^ These results are consistent with prior research that suggests metabolic diagnoses may be a factor contributing to suicide risk.^101–102^

At a macro level, while relatively few studies of functional rare genetic variation have been done in suicide risk, there is convergence between the results of this study and previous work regarding evidence of association with neuropsychiatric, neurodegenerative, inflammatory, and immune risks, both for suicide death^854, 55, 85, 103^ and for suicidal thoughts and behaviors.^22^ This convergence appears to transcend differences in study designs, types of data used, and ascertainment.

## Study Limitations and Future Directions

Although we used an internal replication approach, external replication in independent cohorts is essential to confirm findings, though the limited availability of WGS datasets in individuals who died by suicide present ongoing replication challenges. The data in our study was also enriched for younger age at death, bipolar disorder, and familial risk. While this strategy may have increased power to detect genetic signals, generalizability may have been lessened. Most of our suicide death cohort was of European ancestry, which may limit the generalizability of the results in other populations. Further studies in diverse populations are necessary to validate these findings across different genetic backgrounds.

Additionally, work will be needed to fully characterize the biological impacts of these deletions on gene expression and function. Associations with demographic and clinical data will also require replication, larger samples, and study designs focused on variation across implicated genes aggregated across gene pathways. Next steps will also include broader investigations of other types of genomic variation, including non-coding deletions, other structural variant types (e.g., duplications, inversions), and assessment of putatively functional single nucleotide variants (SNVs). Importantly, future work must also explore interactions among rare variants as well as rare variant interactions with common polygenic backgrounds, demographic attributes, clinical characteristics, and/or environmental exposures.

## Conclusions

This work further advances our understanding of the genetic factors contributing to suicide risk by examining the role of rare, potentially functional intragenic deletions. Our findings implicate diverse mechanisms of risk and suggest the importance of integrating structural variant analysis with existing knowledge from other genetic and non-genetic studies. As the field progresses, further research and replication in independent cohorts will be essential to confirm and extend these results, to understand the underlying biological mechanisms, and to understand demographic, clinical, and environmental contexts interacting with biological risks. Though considerable additional work will be required to identify other rare risk variants and additional complex genetic interaction effects accounting for the missing heritability leading to suicide risk, this knowledge may provide a first step in helping determine subsets of individuals with specific biological risks, and may enable identification of potential drug targets. As part of a complex risk landscape, rare genetic variation plays an essential role in increasing the understanding of complex and heterogenous biological risk mechanisms. However, it is important to emphasize that deletions do not deterministically confer suicide mortality risk, but rather represent only one component within a complex set of interacting genetic and environmental risk factors.

## Supplementary Material

Supplementary information is available at MP’s website.

Supplementary Files

This is a list of supplementary files associated with this preprint. Click to download.

- SupplementalTables16MAY2026.docx
- SupplementDeletionValidationsPCR.pdf
- SupplementalInformation26APR2026.docx
- SupplementDeletionValidationsTaqman.pdf
- SupplementSamplots.pdf

## Figures and Tables

**Table 1:** Demographic characteristics of 1,054 Utah suicide deaths with WGS data.

Cohort	N with WGS	% female	Avg. age at death in years (sd)	% European ancestry	N with bipolar disorder
Cohort 1	670	24.25%	28.87 (sd = 13.08)	97.72%	145
Cohort 2	384	34.12%	37.84 (sd = 12.21)	98.58%	180
All suicides with WGS	1,054	27.84%	32.13 (sd = 13.47)	98.10%	325

Note. Demographic age and sex data were computed from the suicide cohort with verified health records data (N = 1,049).

**Table 2:** Manually validated deletions present in at least two suicides in each of the two suicide death cohorts and showing > two-fold increase in frequency over jointly processed control sequence data.

Chr	Start (hg38)	End	Length	Gene	Frequency, jointly called controls^1^	Frequency, suicide deaths	Fold increase in suicides^2^	p-value^3^	*Frequency, in non-neuro GnomAD* ^4^
22	37019286	37024652	−5366	*MPST*	0.000784	0.00381	4.86	3.12E-04	*0.00089*
17	29056851	29058075	−1224	*PIPOX*	0.000784	0.00381	4.86	3.12E-04	*0.00102*
2	229931901	229954572	−22671	*FBXO36*	0.000876	0.00333	3.81	2.88E-03	*0.00102*
12	25494303	25501878	−7575	*LMNTD1*	0.002393	0.00905	3.78	1.44E-06	*Not reported*
10	96521588	96521862	−274	*TM9SF3*	0.001429	0.00476	3.33	1.09E-03	*0.00168*
19	12292303	12294048	−1745	*ZNF44*	0.002443	0.00762	3.12	8.99E-05	*0.00294*
20	62294459	62295274	−815	*OSBPL2*	0.001405	0.00429	3.05	3.39E-03	*0.00102*
20	41184282	41184388	−106	*ZHX3*	0.001106	0.00333	3.01	9.79E-03	*0.00132*
1	86562531	86573603	−11072	*CLCA4*	0.002291	0.00571	2.49	4.02E-03	*0.00240*
16	27325117	27339881	−14764	*IL4R*	0.003522	0.00810	2.30	1.66E-03	*Not reported*
16	83162542	83176121	−13579	*CDH13*	0.004146	0.00857	2.07	3.75E-03	*0.00450*

Notes. Listed frequencies in suicides are of deletions that were manually validated. A total of 25 deletions in suicides were prioritized with requirements of: meeting quality, length, and frequency criteria to minimize false positive occurrences (see text); and occurrence in at least two suicides in the initial cohort of 670 suicides and at least two suicides in the second cohort of 384 suicides (to increase robustness).

1 Jointly processed control data was chosen to match for ancestry from three sources: N = 622 from the 1000 Genomes Project; N = 512 unrelated individuals from Utah CEPH genetic reference families; N = 96 from Utah study of longevity.

2 Fold increase in suicides indicates the ratio of the frequency in suicides to the frequency in controls.

3 Significance of binomial tests comparing suicide deaths to jointly called controls. Listed p-values listed were all significant at a false discovery rate (FDR) < 0.05.

4 Frequencies are given for comparison only, as data were not jointly processed. GnomAD is the Genome Aggregation Database; the “non-neuro” subset of non-Finnish European ancestry in GnomAD v2.1 (N = 16,684) omits data from individuals with neuropsychiatric and neurological conditions.

**Table 3:** Additional characterizations of deletion regions and implicated genes.

Gene implicated by deletion	GTEx expression of SNPs within deletion regions^1^	GWAS Catalog associations	DGIdb gene-drug interactions	DRUGBANK transcriptomics
*MPST*	No del SNPs overlap with GTEx	Hemoglobin, gut microbiome, metabolite levels, putamen	DGIdb: Thyroxine (thyroid hormone levels)	Upregulated: Selenium (found in supplements; important for prevention of cellular damage by free radicals); Vitamin E (vitamin deficiency) Downregulated: Acetaminophen (analgesic; pain, fever, allergies); Irinotecan (mitochondrial inhibitor; cancer)
*PIPOX*	4 SNPs, adrenal gland expression	MDD, Depression, AD, BMI, accelerated aging, language, smoking heaviness, smoking initiation	None	Upregulated: Belinostat (histone deacetylase inhibitor; cancer); Estradiol (estrogen; menopause); Entinostat (histone deacetylase inhibitor; cancer); Ethinylstradiol (estrogen; menopause, acne); Genistein (isoflavonoid; cardiovascular disease); Panobinostat (histone deacetylase inhibotor; cancer); Valproic acid* (antiepileptic; bipolar disorder, depression, mania, migraine, seizures) Downregulated: Cyclosporine+ (anti-inflammatory; arthritis, other immune conditions); Quercetin (natural favonoid; antioxidant); Theophylline (adenosine antagonist; asthma); Tretinoin (retinoid; acne, skin disorders);
*FBXO36*	19 SNPs, 4 with brain expression	Triglycerides, creatinine, fat mass, BMI	None	Upregulated: Vorinostat (histone deacetylase inhibitor; cancer of the immune system) Downregulated: Acetaminophen (analgesic; pain, fever, allergies);
*LMNTD1*	4 SNPs, testis expression	Gut microbiome, monocyte count	DGIdb: Capecitabine (cytotoxic agent, cancer treatment)	Upregulated: Calcitriol (vitamin; hyperparathyroidism)
*TM9SF3*	No del SNPs overlap with GTEx	Bone density	None	Downregulated: Acetaminophen (analgesic; pain, fever, allergies); Copper (transition metal; nutritional supplements); Cyclosporine+ (anti-inflammatory; arthritis, other immune conditions); Valproic acid* (antiepileptic; bipolar disorder, depression, mania, migraine, seizures)
*ZNF44*	4 SNPs, 3 expressed in brain	Alcoholic liver disease	None	Upregulated: Cyclosporine+ (anti-inflammatory; arthritis, other immune conditions) Downregulated: Methotrexate (thymidylate synthesis; cancer, arthritis); Quercetin (natural flavonoid; antioxidant); Valproic acid* (antiepileptic; bipolar disorder, depression, mania, migraine, seizures); Vitamin E (vitamin deficiency)
*OSBPL2*	1 SNP expressed in testis	Bipolar, platelet volume, nicotine dependence, reticulocyte fraction, cortical folding, educational attainment	None	Upregulated: Bortezomib (proteasome inhibitor; cancer); Cyclosporine+ (anti-inflammatory; arthritis, other immune conditions); Valproic acid* (antiepileptic; bipolar disorder, depression, mania, migraine, seizures) Downregulated: Methotrexate (thymidylate synthesis; cancer, arthritis)
*ZHX3*	No del SNPs overlap with GTEx	Unipolar depression, daytime napping, sleep duration, MDD, depressive symptoms, drinks per week, migraine, triglycerides, cholesterol, CAD, BMI, waist-hip ratio	None	Upregulated: Copper (transition metal; nutritional supplements); Disulfiram (aldehyde dehydrogenase inhibitor; alcohol addiction); Methotrexate (thymidylate synthesis; cancer, arthritis); Valproic acid* (antiepileptic; bipolar disorder, depression, mania, migraine, seizures) Downregulated: Cyclosporine+ (anti-inflammatory; arthritis, other immune conditions)
*CLCA4*	11 SNPs, all associated with brain expression	IgG glycosylation, macrophage inflammatory protein 1b levels	None	Downregulated: silicon dioxide (found in supplements; respiratory toxicity)
*IL4R*	27 SNPs, 17 with brain expression	Asthma, allergy, C-reactive protein, respiratory diseases, IgE levels, eosinophil counts	DGIdb: Sunitinib (antineoplastic agent; cancer); Cintredekin Besudotox (antineoplastic agent; cancer); Pitrakinra (IL4 and IL13 inhibitor; asthma); Bizaxofusp (antineoplastic agent, cancer); Romilkimab (IgG4 antibody; fibrous diseases); Dupilumab (monoclonal antibody, asthma, COPD, dermatitis)	Upregulated: Estradiol (estrogen; menopause); Cyclosporine+ (anti-inflammatory; arthritis, other immune conditions); Cisplatin (chemotherapy; cancer); Diethylstibestrol (estrogen receptor; menopause); Calcitriol (vitamin; hypoparathyroidism); Genistein (estrogen receptor; menopause); Imatnib (kinase inhibitor; cancer); Methotrexate (thymidylate synthesis; cancer, arthritis); Selenium (found in supplements; important for prevention of cellular damage by free radicals); high cholesterol, stroke, CVD); Testosterone (androgen; low testosterone, breast cancer); Vitamin E (vitamin deficiency); Zinc (vitamin deficiency) Downregulated: Arsenic trioxode (nuclear factor kappa-b inhibitor; cancer); Belinostat (histone deacetylase inhibitor; cancer); Dexamethasone (glucocorticoid receptor; immune diseases); Fenretinide (synthetic retinoid; cancer); Quercetin (natural favonoid; antioxidant); Simvistatin (hydroxy-methylglutaryl-CoA reductase inhibitor;
*CDH13*	No del SNPs overlap with GTEx	ADHD, smoking initiation, clozapine response, amphetamine response, SZ, AD onset age, educational attainment, cognitive ability, CAD, blood pressure, adiponectin levels, COPD treatment response, BMI, antibody response to COVID-19 vaccine	DGIdb: Tritiated dihydrotetrabenazine (radioligand, used to assess binding of vesicular monoamine transporter-VMAT2 in studies of neurodegenration or psychiatric disorders); Ketanserin (antihypertensive; hypertension); Dextroamphetamine sulfate (CNS stimulant; ADHD, narcolepsy, cognitive dysfunction); Reserpine (antihypertensive/ antipsychotic; hypertension, psychotic agitation); Aspirin (NSAID); Iodovinyltetrabenazine (radioligand VMAT2 binding agent; used in PET imaging for neurodenerative disorders); Tetrabenazine (VMAT2 inhibitor, Huntington's chorea, Tourette, tardive diskinesia); Perphenazine (antipsychotic, dopamine blocker; schizophrenia); [125]7-Azido-8-iodoketanserine (photoaffinity probe; used to study VMAT 2 and serotonin receptors); Clopidogrel (antiplatelet; clotting disorders, stroke); Sorafenib (multi-kinase inhibitor; cancer); Deutetrabenazine (tardive diskinesia, chorea in HD, NOTE: may increase suicidality); Carbon-11 labeled dihydrotetrabenazine (PET radioligand; used to image brain VMAT2)	Upregulated: Tretinoin (retinoid; cancer, skin conditions) Downregulated: Acetylcysteine (glutothione synthetase; lung conditions, acetaminophen overdose); Epigallocatechin gallate (hypertension, diabetic neuropathy); Folic acid (found in supplements; folate deficiency, anemia)

1 Further detail of SNP names, p-values, and GTEx-associated tissues appears in Supplemental Table S3. See Supplemental information for a listing of GWAS Catalog references.

* Valproic acid is a histone deacetylase (HDAC) inhibitor known to alter expression of many genes involved in both inflammation and neurodevelopment.^81^

+ Cyclosporine alters expression of many genes across a broad network of immune pathways.^97^

## References

[1] Garnett MF, Curtin SC. Suicide Mortality in the United States, 2002–2022. NCHS Data Brief. 2024 Sep; (509):10.15620/cdc/160504. PMC11615958 39392858

[2] McGuffin P, Marusic A, Farmer A. What can psychiatric genetics offer suicidology? Crisis. 2001;22(2):61–5. 11727895 10.1027//0227-5910.22.2.61

[3] Pedersen NL, Fiske A. Genetic influences on suicide and nonfatal suicidal behavior: twin study findings. Eur Psychiatry. 2010 Jun;25(5):264–7. 20444580 10.1016/j.eurpsy.2009.12.008 PMC4537645

[4] Statham DJ, Heath AC, Madden PA, Bucholz KK, Bierut L, Dinwiddie SH, Slutske WS, Dunne MP, Martin NG. Suicidal behaviour: an epidemiological and genetic study. Psychol Med. 1998 Jul;28(4):839–55. 9723140 10.1017/s0033291798006916

[5] Kimbrel NA, Ashley-Koch AE, Qin XJ, Lindquist JH, Garrett ME, Dennis MF, Hair LP, Identification of Novel, Replicable Genetic Risk Loci for Suicidal Thoughts and Behaviors Among US Military Veterans. JAMA Psychiatry. 2023 Feb 1;80(2):135–145. 36515925 10.1001/jamapsychiatry.2022.3896 PMC9857322

[6] Docherty AR, Mullins N, Ashley-Koch AE, Qin X, Coleman JRI, Shabalin A, Kang J, GWAS Meta-Analysis of Suicide Attempt: Identification of 12 Genome-Wide Significant Loci and Implication of Genetic Risks for Specific Health Factors. Am J Psychiatry. 2023 Oct 1;180(10):723–738. 37777856 10.1176/appi.ajp.21121266 PMC10603363

[7] Li QS, Shabalin AA, DiBlasi E, Gopal S, Canuso CM; FinnGen, International Suicide Genetics Consortium; Palotie A, Drevets WC, Docherty AR, Coon H. Genome-wide association study meta-analysis of suicide death and suicidal behavior. Mol Psychiatry. 2023 Feb;28(2):891–900. 36253440 10.1038/s41380-022-01828-9 PMC9908547

[8] Mullins N, Kang J, Campos AI, Coleman JRI, Edwards AC, Galfalvy H, Levey DF, Dissecting the Shared Genetic Architecture of Suicide Attempt, Psychiatric Disorders, and Known Risk Factors. Biol Psychiatry. 2022 Feb 1;91(3):313–327. 34861974 10.1016/j.biopsych.2021.05.029 PMC8851871

[9] Colbert SMC, Koromina M, Hatoum AS, Stephenson M, Edwards AC, Johnson EC, Qin X, Genome-wide association studies identify 77 loci for suicidality and provide novel biological insights, MedRxiv,2025.10.22.25338076; doi: 10.1101/2025.10.22.25338076

[10] He J, Cabrera-Mendoza B, Qiu D, Davtian D, Mao Z, Chen Q, Penichet EN, Zhang Q, Karaca S, Polimanti R. Multivariate genome-wide association study of suicidal behaviors in >1.7 million individuals of diverse population descents. medRxiv [Preprint]. 2025 Dec 16:2025.12.15.25342298. doi: 10.64898/2025.12.15.25342298.

[11] Chesney E, Goodwin GM, Fazel S. Risks of all-cause and suicide mortality in mental disorders: a meta-review. World Psychiatry. 2014;13(2):153–60. 24890068 10.1002/wps.20128 PMC4102288

[12] Favril L, Yu RQ, Geddes JR, Fazel S. Individual-level risk factors for suicide mortality in the general population: an umbrella review. Lancet Public Health 2023; 8: e868–77 37898519 10.1016/S2468-2667(23)00207-4 PMC10932753

[13] Xiao Y, Bi K, Yip PS, Cerel J, Brown TT, Peng Y, Pathak J, Mann JJ. Decoding Suicide Decedent Profiles and Signs of Suicidal Intent Using Latent Class Analysis. JAMA Psychiatry. 2024 Mar 20. 10.1001/jamapsychiatry.2024.0171 PMC10955339 38506817

[14] Coon H, Shabalin AA, Monson ET, DiBlasi E, Han S, Baird LM, Kaufman EA, Tharp D, Staley MJ, Yu Z, Li QS, Colbert SM, Bakian AV, Docherty AR, McIntosh AM, Whalley HC, Amaro D, Crockett DK, Mullins N, Keeshin BR. Genetic Liabilities to Neuropsychiatric Conditions in Suicide Deaths With No Prior Suicidality. JAMA Netw Open. 2025 Oct 1;8(10):e2538204. 41114977 10.1001/jamanetworkopen.2025.38204 PMC12538367

[15] Coon H, Shabalin AA, DiBlasi E, Monson ET, Han S, Kaufman EA, Chen D, Kious B, Molina N, Yu Z, Staley MJ, Crockett DK, Colbert SM, Mullins N, Bakian AV, Docherty AR, Keeshin BR. Absence of nonfatal suicidal behavior preceding suicide death reveals differences in clinical risks. Psychiatry Res. 2025 May;347:116391. 40020535 10.1016/j.psychres.2025.116391 PMC11976895

[16] Althoff RR, Hudziak JJ, Willemsen G, Hudziak V, Bartels M, Boomsma DI. Genetic and environmental contributions to self-reported thoughts of self-harm and suicide. Am J Med Genet B: Neuropsychiat Genet 2011 159B(1):120–7. 10.1002/ajmg.b.32010 PMC3254180 22162437

[17] McGirr A, Alda M, Seguin M, Cabot S, Lesage A, Turecki G (2009) Familial aggregation of suicide explained by cluster B traits: a three-group family study of suicide controlling for major depressive disorder. Am J Psychiatry 166(10):1124–34. 19755577 10.1176/appi.ajp.2009.08111744

[18] Kim CD, Seguin M, Therrien N, Riopen G, Chawky N, Sesage AD, Turecki G (2005) Familial aggregation of suicidal behavior: a family study of male suicide completers from the general population. Am J Psychiat 162:1017–1019. 15863812 10.1176/appi.ajp.162.5.1017

[19] Brent DA, Mann JJ (2005) Family genetic studies, suicide, and suicidal behavior. Am J Med Genet. 133C:13–24. 15648081 10.1002/ajmg.c.30042

[20] Génin E. Missing heritability of complex diseases: case solved? Hum Genet. 2020 Jan;139(1):103–113. 31165258 10.1007/s00439-019-02034-4

[21] De Deyn L, Sleegers K. The impact of rare genetic variants on Alzheimer disease. Nat Rev Neurol. 2025 Mar;21(3):127–139. 39905212 10.1038/s41582-025-01062-1

[22] Garrett ME, Dennis MF, Bourassa KJ; VA Mid-Atlantic MIRECC Workgroup; Beckham JC, Kimbrel NA, Ashley-Koch AE. Whole exome sequencing analysis of suicidal thoughts and behaviors in a veteran cohort implicates inflammatory pathways and genes previously associated with psychiatric and neurodegenerative diseases. J Affect Disord. 2026 Jan 15;393(Pt A):120301. 40945769 10.1016/j.jad.2025.120301 PMC13244272

[23] Owen MJ, Williams NM. Explaining the missing heritability of psychiatric disorders. World Psychiatry. 2021 Jun;20(2):294–295. 34002520 10.1002/wps.20870 PMC8129850

[24] Ho SS, Urban AE, Mills RE. Structural variation in the sequencing era. Nat Rev Genet. 2020 Mar;21(3):171–189. 31729472 10.1038/s41576-019-0180-9 PMC7402362

[25] Weischenfeldt J, Symmons O, Spitz F, Korbel JO. Phenotypic impact of genomic structural variation: insights from and for human disease. Nat Rev Genet. 2013 Feb;14(2):125–38. 23329113 10.1038/nrg3373

[26] Andreassen OA, Hindley GFL, Frei O, Smeland OB. New insights from the last decade of research in psychiatric genetics: discoveries, challenges and clinical implications. World Psychiatry. 2023 Feb;22(1):4–24. 36640404 10.1002/wps.21034 PMC9840515

[27] Coon H, Darlington TM, DiBlasi E, Callor WB, Ferris E, Fraser A, Yu Z, Genome-wide significant regions in 43 Utah high-risk families implicate multiple genes involved in risk for completed suicide. Mol Psychiatry. 2020 Nov;25(11):3077–3090. 30353169 10.1038/s41380-018-0282-3 PMC6478563

[28] Chiang C, Layer RM, Faust GG, Lindberg MR, Rose DB, Garrison EP, Marth GT, Quinlan AR, Hall IM. SpeedSeq: ultra-fast personal genome analysis and interpretation. Nat Methods. 2015 Oct;12(10):966–8. 26258291 10.1038/nmeth.3505 PMC4589466

[29] Coon H, Shabalin A, Bakian AV, DiBlasi E, Monson ET, Kirby A, Chen D, Extended familial risk of suicide death is associated with younger age at death and elevated polygenic risk of suicide. Am J Med Genet B Neuropsychiatr Genet. 2022 Apr;189(3–4):60–73. 35212135 10.1002/ajmg.b.32890 PMC9149029

[30] Miller JN, Black DW. Bipolar Disorder and Suicide: a Review. Curr Psychiatry Rep. 2020 Jan 18;22(2):6. 31955273 10.1007/s11920-020-1130-0

[31] Byrska-Bishop M, Evani US, Zhao X, Basile AO, Abel HJ, Regier AA, Corvelo A, High-coverage whole-genome sequencing of the expanded 1000 Genomes Project cohort including 602 trios. Cell. 2022 Sep 1;185(18):3426–3440.e19. 36055201 10.1016/j.cell.2022.08.004 PMC9439720

[32] Dausset J, Cann H, Cohen D, Lathrop M, Lalouel JM, White R. Centre d'etude du polymorphisme humain (CEPH): collaborative genetic mapping of the human genome. Genomics. 1990 Mar;6(3):575–7. 2184120 10.1016/0888-7543(90)90491-c

[33] Tschanz JT, Corcoran C, Skoog I, Khachaturian AS, Herrick J, Hayden KM, Welsh-Bohmer KA, Dementia: the leading predictor of death in a defined elderly population: the Cache County Study. Neurology. 2004 Apr 13;62(7):1156–62. 15079016 10.1212/01.wnl.0000118210.12660.c2

[34] Purcell S, Neale B, Todd-Brown K, Thomas L, Ferreira MA, Bender D, Maller J, Sklar P, de Bakker PI, Daly MJ, Sham PC. PLINK: a tool set for whole-genome association and population-based linkage analyses. Am J Hum Genet. 2007 Sep;81(3):559–75. 17701901 10.1086/519795 PMC1950838

[35] Genovese G. kgp2anc: A protocol to estimate ancestry from the main ethnicities (European, African, East Asian, Native American, South Asian) using principal components analysis together with individuals from the 1000 Genomes project phase 3 starting from raw Illumina genotype array data. Github 2025, code and documentation at https://github.com/freeseek/kgp2anc.

[36] Aldana R, Freed D. Data Processing and Germline Variant Calling with the Sentieon Pipeline. Methods Mol Biol. 2022;2493:1–19. 35751805 10.1007/978-1-0716-2293-3_1

[37] Depristo MA, Banks E, Poplin R, Garimella KV, Maguire JR, Hartl C, Philippakis AA, A framework for variation discovery and genotyping using next-generation DNA sequencing data. Nature Genetics, 2011 43(5). 10.1038/ng.806 PMC3083463 21478889

[38] Van der Auwera GA, Carneiro MO, Hartl C, Poplin R, del Angel G, Levy-Moonshine A, Jordan T, From fastQ data to high-confidence variant calls: The genome analysis toolkit best practices pipeline. Current Protocols in Bioinformatics, 2013 SUPL.43. 10.1002/0471250953.bi1110s43 PMC4243306 25431634

[39] Nicholas TJ, Al-Sweel N, Farrell A, Mao R, Bayrak-Toydemir P, Miller CE, Bentley D, (2022). Comprehensive variant calling from whole-genome sequencing identifies a complex inversion that disrupts ZFPM2 in familial congenital diaphragmatic hernia. Molecular Genetics and Genomic Medicine, 2022 10(4). 10.1002/mgg3.1888 PMC9000945 35119225

[40] Li H, Durbin R. Fast and accurate short read alignment with Burrows-Wheeler transform. Bioinformatics, 2009 25(14). 10.1093/bioinformatics/btp324 PMC2705234 19451168

[41] Chen X, Schulz-Trieglaff O, Shaw R, Barnes B, Schlesinger F, Källberg M, Cox AJ, Manta: Rapid detection of structural variants and indels for germline and cancer sequencing applications. Bioinformatics, 2016 32(8). 10.1093/bioinformatics/btv710 26647377

[42] Pedersen B. S., & Quinlan A. R. (2019). Duphold: Scalable, depth-based annotation and curation of high-confidence structural variant calls. GigaScience, 8(4). 10.1093/gigascience/giz040 PMC6479422 31222198

[43] Nicholas TJ, Cormier MJ, Quinlan AR. Annotation of structural variants with reported allele frequencies and related metrics from multiple datasets using SVAFotate. 2022 BMC Bioinformatics 23, 490. 36384437 10.1186/s12859-022-05008-y PMC9670370

[44] Collins R. L., Brand H., Karczewski K. J., Zhao X., Alföldi J., Francioli L. C., Khera A. V., Lowther C., Gauthier L. D., Wang H., Watts N. A., Solomonson M., O’Donnell-Luria A., Baumann A., Munshi R., Walker M., Whelan C. W., Huang Y., Brookings T., … Talkowski M. E. (2020). A structural variation reference for medical and population genetics. Nature, 581(7809). 10.1038/s41586-020-2287-8 PMC7334194 32461652

[45] Abel H. J., Larson D. E., Regier A. A., Chiang C., Das I., Kanchi K. L., Layer R. M., Neale B. M., Salerno W. J., Reeves C., Buyske S., Abecasis G. R., Appelbaum E., Baker J., Banks E., Bernier R. A., Bloom T., Boehnke M., Boerwinkle E., … Hall I. M. (2020). Mapping and characterization of structural variation in 17,795 human genomes. Nature, 583(7814). 10.1038/s41586-020-2371-0 PMC7547914 32460305

[46] Pedersen BS, Brown JM, Dashnow H, Wallace AD, Velinder M, Tristani-Firouzi M, Schiffman JD. Effective variant filtering and expected candidate variant yield in studies of rare human disease. Npj Genomic Medicine, 2021 6(1). 10.1038/s41525-021-00227-3 PMC8282602 34267211

[47] Gudmundsson S, Singer-Berk M, Watts NA, Phu W, Goodrich JK, Solomonson M, Genome Aggregation Database Consortium, Variant interpretation using population databases: Lessons from gnomAD. Hum Mutation 2021 Dec 02, 10.1002/humu.24309. PMC9160216 34859531

[48] Belyeu JR, Chowdhury M, Brown J, Pedersen BS, Cormier MJ, Quinlan AR, Layer RM. Samplot: a platform for structural variant visual validation and automated filtering. Genome Biology, 2021 22(1). 10.1186/s13059-021-02380-5 PMC8145817 34034781

[49] Cannon M, Stevenson J, Stahl K, Basu R, Coffman A, Kiwala S, McMichael JF, DGIdb 5.0: rebuilding the drug-gene interaction database for precision medicine and drug discovery platforms. Nucleic Acids Research, 2024 52(D1). 10.1093/nar/gkad1040 PMC10767982 37953380

[50] Knox C, Wilson M, Klinger CM, DrugBank 6.0: the DrugBank Knowledgebase for 2024. Nucleic Acids Res. 2024 Jan 5;52(D1):D1265–D1275. 37953279 10.1093/nar/gkad976 PMC10767804

[51] Sollis E, Mosaku A, Abid A, Buniello A, Cerezo M, Gil L, Groza T, The NHGRI-EBI GWAS Catalog: knowledgebase and deposition resource. Nucleic Acids Research, 2023 51(1 D). 10.1093/nar/gkac1010 PMC9825413 36350656

[52] Denny JC, Ritchie MD, Basford MA, Pulley JM, Bastarache L, Brown-Gentry K, Wang D, PheWAS: Demonstrating the feasibility of a phenome-wide scan to discover gene-disease associations. Bioinformatics, 2010 26(9). 10.1093/bioinformatics/btq126 PMC2859132 20335276

[53] Shuey MM, Stead WW, Aka I, Barnado AL, Bastarache JA, Brokamp E, Campbell M, Next-generation phenotyping: introducing phecodeX for enhanced discovery research in medical phenomics. Bioinformatics, 2023 39(11). 10.1093/bioinformatics/btad655 PMC10627409 37930895

[54] Coon H, Darlington TM, DiBlasi E, Callor WB, Ferris E, Fraser A, Yu Z, Genome-wide significant regions in 43 Utah high-risk families implicate multiple genes involved in risk for completed suicide. Mol Psychiatry. 2020 Nov;25(11):3077–3090. 30353169 10.1038/s41380-018-0282-3 PMC6478563

[55] DiBlasi E, Shabalin AA, Monson ET, Keeshin BR, Bakian AV, Kirby AV, Ferris E, Rare protein-coding variants implicate genes involved in risk of suicide death. Am J Med Genet B Neuropsychiatr Genet. 2021 Dec;186(8):508–520. 34042246 10.1002/ajmg.b.32861 PMC9292859

[56] Bonk S., Eszlari N., Kirchner K., Gezsi A., Garvert L., Koukkanen M., Cano I., Grabe H. J., Antal P., Juhasz G., & Van der Auwera S. (2024). Impact of gene-by-trauma interaction in MDD-related multimorbidity clusters. Journal of Affective Disorders, 359, 382–391. 38806065 10.1016/j.jad.2024.05.126

[57] Ide M, Ohnishi T, Toyoshima M, Balan S, Maekawa M, Shimamoto-Mitsuyama C, Iwayama Y, Ohba H, Watanabe A, Ishii T, Shibuya N, Kimura Y, Hisano Y, Murata Y, Hara T, Morikawa M, Hashimoto K, Nozaki Y, Toyota T, Wada Y, Tanaka Y, Kato T, Nishi A, Fujisawa S, Okano H, Itokawa M, Hirokawa N, Kunii Y, Kakita A, Yabe H, Iwamoto K, Meno K, Katagiri T, Dean B, Uchida K, Kimura H, Yoshikawa T. Excess hydrogen sulfide and polysulfides production underlies a schizophrenia pathophysiology. EMBO Mol Med. 2019 Dec;11(12):e10695. 31657521 10.15252/emmm.201910695 PMC6895609

[58] Wang L, Sha H, He X, Xie Y, Deng J, Chen J, Li G, Yang J. Neonatal IL-4 Over-Exposure is Accompanied by Macrophage Accumulation in Dura Mater After Instant Anti-inflammatory Cytokine Response in CSF. Cell Mol Neurobiol. 2024 Feb 5;44(1):18. 38315435 10.1007/s10571-023-01451-4 PMC10844484

[59] Zhang J, Rong P, Zhang L, He H, Zhou T, Fan Y, Mo L, Zhao Q, Han Y, Li S, Wang Y, Yan W, Chen H, You Z. IL4-driven microglia modulate stress resilience through BDNF-dependent neurogenesis. Sci Adv. 2021 Mar 17;7(12):eabb9888. 10.1126/sciadv.abb9888 PMC7968840 33731342

[60] Nisbet L, Wu Y, Adams M, Lynall ME, Hjerling-Leffler J; Psychiatric Genomics Consortium Functional Genomics Working Group; Wray NR, McIntosh AM, Shen X. Integrating Multi-Omics Summary Data Identifies Candidate Molecular Mechanisms for Major Depression. Biol Psychiatry. 2025 Dec 15:S0006-3223(25)01675-0. 10.1016/j.biopsych.2025.11.024 PMC13527628 41407005

[61] Salatino-Oliveira A, Genro JP, Polanczyk G, Zeni C, Schmitz M, Kieling C, Anselmi L, Cadherin-13 gene is associated with hyperactive/impulsive symptoms in attention/deficit hyperactivity disorder. American Journal of Medical Genetics, Part B: Neuropsychiatric Genetics, 2015 168(3). 10.1002/ajmg.b.32293 25739828

[62] He H, Guzman RE, Cao D, Sierra-Marquez J, Yin F, Fahlke C, Peng J, Stauber T. The molecular and phenotypic spectrum of CLCN4-related epilepsy. Epilepsia, 2021 62(6). 10.1111/epi.16906 33951195

[63] Bassuk AG, Geraghty E, Wu S, Mullen SA, Berkovic SF, Scheffer IE, Mefford HC. Deletions of 16p11.2 and 19p13.2 in a family with intellectual disability and generalized epilepsy. American Journal of Medical Genetics, Part A, 2013 161(7). 10.1002/ajmg.a.35946 PMC4169108 23686817

[64] Kirby AV, Bakian AV, Zhang Y, Bilder DA, Keeshin BR, Coon H. A 20-year study of suicide death in a statewide autism population. Autism Res. 2019 Apr;12(4):658–666. 30663277 10.1002/aur.2076 PMC6457664

[65] Vialle RA, de Paiva Lopes K, Li Y, Ng B, Schneider JA, Buchman AS, Wang Y, Farfel JM, Barnes LL, Wingo AP, Wingo TS, Seyfried NT, De Jager PL, Gaiteri C, Tasaki S, Bennett DA. Structural variants linked to Alzheimer's disease and other common age-related clinical and neuropathologic traits. Genome Med. 2025 Mar 4;17(1):20. 40038788 10.1186/s13073-025-01444-6 PMC11881306

[66] Igata T., Tanaka H., Etoh K., Hong S., Tani N., Koga T., & Nakao M. (2022). Loss of the transcription repressor ZHX3 induces senescence-associated gene expression and mitochondrial-nucleolar activation. PLoS ONE, 17(1 January). 10.1371/journal.pone.0262488 PMC8794122 35085309

[67] Li W, Shan B, Cheng X, He H, Qin J, Zhao H, Tian M, Zhang X, Jin G. circRNA Acbd6 promotes neural stem cell differentiation into cholinergic neurons via the miR-320–5p-Osbpl2 axis. J Biol Chem. 2022 Apr;298(4):101828. 35305988 10.1016/j.jbc.2022.101828 PMC9018392

[68] Whitehouse PJ, Price DL, Struble RG, Clark AW, Coyle JT, Delon MR. Alzheimer's disease and senile dementia: loss of neurons in the basal forebrain. Science. 1982 Mar 5;215(4537):1237–9. 7058341 10.1126/science.7058341

[69] Kalidasan V, Suresh D, Zulkifle N, Hwei YS, Kok Hoong L, Rajasuriar R, Theva Das K. Investigating D-Amino Acid Oxidase Expression and Interaction Network Analyses in Pathways Associated With Cellular Stress: Implications in the Biology of Aging. Bioinform Biol Insights. 2024 Feb 28;18:11779322241234772. 10.1177/11779322241234772 PMC10903195 38425413

[70] Prata DP, Costa-Neves B, Cosme G, Vassos E. Unravelling the genetic basis of schizophrenia and bipolar disorder with GWAS: A systematic review. J Psychiatr Res. 2019 Jul;114:178–207. 31096178 10.1016/j.jpsychires.2019.04.007

[71] Hawi Z, Tong J, Dark C, Yates H, Johnson B, Bellgrove MA. The role of cadherin genes in five major psychiatric disorders: A literature update. Am J Med Genet B Neuropsychiatr Genet. 2018 Mar;177(2):168–180. 28921840 10.1002/ajmg.b.32592

[72] Børglum AD, Demontis D, Grove J, Pallesen J, Hollegaard MV, Pedersen CB, Hedemand A, Genome-wide study of association and interaction with maternal cytomegalovirus infection suggests new schizophrenia loci. Mol Psychiatry. 2014 Mar;19(3):325–33. 23358160 10.1038/mp.2013.2 PMC3932405

[73] Coleman JRI, Gaspar HA, Bryois J, Byrne EM, Forstne AJ, Holmans PA, de Leeuw CA, The Genetics of the Mood Disorder Spectrum: Genome-wide Association Analyses of More Than 185,000 Cases and 439,000 Controls. Biological Psychiatry, 2020 88(2). 10.1016/j.biopsych.2019.10.015 PMC8136147 31926635

[74] Fabbri C., Kasper S., Kautzky A., Bartova L., Dold M., Zohar J., Souery D., Montgomery S., Albani D., Raimondi I., Dikeos D., Rujescu D., Uher R., Lewis C. M., Mendlewicz J., & Serretti A. (2019). Genome-wide association study of treatment-resistance in depression and meta-analysis of three independent samples. British Journal of Psychiatry, 214(1). 10.1192/bjp.2018.256 30468137

[75] Mullins N, Forstner AJ, O’Connell KS, Coombes B, Coleman JRI, Qiao Z, Als TD, Genome-wide association study of more than 40,000 bipolar disorder cases provides new insights into the underlying biology. Nature Genetics, 2021 53(6). 10.1038/s41588-021-00857-4 PMC8192451 34002096

[76] Frank S, Testa C, Edmondson MC, Goldstein J, Kayson E, Leavitt BR, Oakes D, O'Neill C, Vaughan C, Whaley J, Gross N, Gordon MF, Savola JM; Huntington Study Group/ARC-HD Investigators and Coordinators. The Safety of Deutetrabenazine for Chorea in Huntington Disease: An Open-Label Extension Study. CNS Drugs. 2022 Nov;36(11):1207–1216. 36242718 10.1007/s40263-022-00956-8 PMC9653309

[77] Qing G, Ye S, Wei B, Yang Y. Real-world safety analysis of deutetrabenazine post-marketing: a disproportionality study leveraging the FDA Adverse Event Reporting System (FAERS) database. BMC Pharmacol Toxicol. 2025 Feb 21;26(1):41. 39985106 10.1186/s40360-025-00872-9 PMC11846250

[78] Liwinski T, Lang UE. Folate and Its Significance in Depressive Disorders and Suicidality: A Comprehensive Narrative Review. Nutrients. 2023 Sep 4;15(17):3859. 37686891 10.3390/nu15173859 PMC10490031

[79] Li J, Wang L, Wang Z, Zhao F, Sun Y, Lu Y, Yang L. Association between suicide attempts and anemia in late-life depression inpatients. BMC Geriatr. 2024 Jan 10;24(1):43. 38200429 10.1186/s12877-023-04649-9 PMC10782764

[80] Mann JJ, Hur K, Lavigne JE, Gibbons RD. Folic acid prescription and suicide attempt prevention: effect of past suicidal behaviour, psychiatric diagnosis and psychotropic medication. BJPsych Open. 2023 Aug 22;9(5):e159. 37605842 10.1192/bjo.2023.549 PMC10486216

[81] Pan LA, Segreti AM, Wrobleski J, Shaw A, Hyland K, Hughes M, Finegold DN, Naviaux RK, Brent DA, Vockley J, Peters DG. Metabolomic disorders: confirmed presence of potentially treatable abnormalities in patients with treatment refractory depression and suicidal behavior. Psychol Med. 2023 Oct;53(13):6046–6054. 36330595 10.1017/S0033291722003233 PMC10520591

[82] Gibbons RD, Hur K, Lavigne JE, Mann JJ. Association Between Folic Acid Prescription Fills and Suicide Attempts and Intentional Self-harm Among Privately Insured US Adults. JAMA Psychiatry. 2022 Nov 1;79(11):1118–1123. 36169979 10.1001/jamapsychiatry.2022.2990 PMC9520442

[83] Doering S, Jonson M, Ernback Schön K, Lindström S, Ehnvall A, Wiktorsson S, Stefenson A, Westrin Å, Waern M. Substance use issues preceding suicide: A Swedish nationwide record review. Drug Alcohol Depend. 2025 Sep 1;274:112736. 40482518 10.1016/j.drugalcdep.2025.112736

[84] Fukuchi M, Nii T, Ishimaru N, Minamino A, Hara D, Takasaki I, Tabuchi A, Tsuda M. Valproic acid induces up- or down-regulation of gene expression responsible for the neuronal excitation and inhibition in rat cortical neurons through its epigenetic actions. Neurosci Res. 2009 Sep;65(1):35–43. 19463867 10.1016/j.neures.2009.05.002

[85] Han S, DiBlasi E, Monson ET, Shabalin AA, Baird L, Chen D, Lamichhane D, Tharp D, Ferris E, Yu Z, Brandon Callor W, Staley MJ, Li QS, Willour V, Crockett DK, Eilbeck K, Bakian AV, Keeshin BR, Okifuji A, Coon H, Docherty AR. Genetic risk of chronic pain conditions associated with risk of suicide death through an integrative analysis of EHR and genomics data. Transl Psychiatry. 2026 Feb 16;16(1):117. 41698884 10.1038/s41398-026-03861-6 PMC12949045

[86] DiBlasi E, Kaufman EA, Webster S, Hagn EE, Shabalin AA, Chen D, Han S, Jawish R, Monson ET, Staley MJ, Keeshin BR, Docherty AR, Bakian AV, Okifuji A, Coon H. Phenome-wide diagnostic comparison among suicide deaths and living individuals with chronic pain diagnoses. BMC Med. 2024 Dec 2;22(1):568. 39617899 10.1186/s12916-024-03794-1 PMC11610288

[87] Yang F, Zheng J, Gao M, Ning L. Genetic mechanisms of pollinosis: interactions between genes and environmental factors. Inflamm Res. 2025 Dec 10;75(1):1. 41372550 10.1007/s00011-025-02150-0

[88] Li W, Shan B, Cheng X, He H, Qin J, Zhao H, Tian M, Zhang X, Jin G. circRNA Acbd6 promotes neural stem cell differentiation into cholinergic neurons via the miR-320–5p-Osbpl2 axis. J Biol Chem. 2022 Apr;298(4):101828. 35305988 10.1016/j.jbc.2022.101828 PMC9018392

[89] Wu Y, Liang S, Zhu H, Zhu Y. Analysis of immune-related key genes in Alzheimer's disease. Bioengineered. 2021 Dec;12(2):9610–9624. 34719321 10.1080/21655979.2021.1999553 PMC8809910

[90] Paulovičová E., Kroneková Z., Paulovičová L., Majerčíková M., & Kronek J. (2021). Cell-mediated immunoreactivity of poly(2-isopropenyl-2-oxazoline) as promising formulation for immunomodulation. Materials, 14(6). 10.3390/ma14061371 PMC7999147 33809040

[91] Vogelaar CF, Mandal S, Lerch S, Birkner K, Birkenstock J, Bühler U, Schnatz A, Fast direct neuronal signaling via the IL-4 receptor as therapeutic target in neuroinflammation. Science Translational Medicine, 2018 10(430). 10.1126/scitranslmed.aao2304 PMC12102061 29491183

[92] Walker A, Mitchell BL, Lin T, Crouse JJ, Albiñana C, Yap CX, Lynall ME, Lind PA, Cipriani A, Byrne EM, Medland SE, Martin NG, Taquet M, Hickie IB, Wray NR. Genetic and Phenotypic Associations With Sustained Antidepressant Use in Major Depressive Disorder. JAMA Psychiatry. 2026 Mar 1;83(3):247–258. 41604195 10.1001/jamapsychiatry.2025.4372 PMC12853290

[93] Kouter K, Šmon J, Videtič Paska A. Epigenetics and immunology: Under-recognized aspects of suicidality. World J Psychiatry. 2025 Sep 19;15(9):107726. 40933164 10.5498/wjp.v15.i9.107726 PMC12417943

[94] Ponce-Regalado MD, Becerril-Villanueva E, Maldonado-García JL, Moreno-Lafont MC, Martínez-Ramírez G, Jacinto-Gutiérrez S, Arreola R, Sánchez-Huerta K, Contis-Montes de Oca A, López-Martínez KM, Bautista-Rodríguez E, Chin-Chan JM, Pavón L, Pérez-Sánchez G. Comprehensive view of suicide: A neuro-immune-endocrine approach. World J Psychiatry. 2025 Feb 19;15(2):98484. 39974471 10.5498/wjp.v15.i2.98484 PMC11758041

[95] Sun S, Liu Q, Wang Z, Huang YY, Sublette ME, Dwork AJ, Rosoklija G, Ge Y, Galfalvy H, Mann JJ, Haghighi F. Brain and blood transcriptome profiles delineate common genetic pathways across suicidal ideation and suicide. Mol Psychiatry. 2024 May;29(5):1417–1426. 38278992 10.1038/s41380-024-02420-z PMC11189724

[96] Lamichhane DK, VanDerslice JA, Lurmann F, Pavlovic NR, Staley MJ, Tharp DS, Peluso A, Byrwa-Hill BM, Zhang Y, Docherty AR, Coon H, Bakian AV. Independent and interactive effects of wet bulb globe temperature and air pollution exposures on suicide mortality. Environ Int. 2026 Feb 21;209:110152. 41774964 10.1016/j.envint.2026.110152 PMC13051550

[97] Bakian AV, Huber RS, Coon H, Gray D, Wilson P, McMahon WM, Renshaw PF. Acute air pollution exposure and risk of suicide completion. Am J Epidemiol. 2015 Mar 1;181(5):295–303. 25673816 10.1093/aje/kwu341 PMC4339389

[98] Reed JC, Prystowsky MB, Nowell PC. Regulation of gene expression in lectin-stimulated or lymphokine-stimulated T lymphocytes. Effects of cyclosporine. Transplantation. 1988 Aug;46(2 Suppl):85S–89S. 3043799 10.1097/00007890-198808001-00016

[99] Riveros-Mckay F, Oliver-Williams C, Karthikeyan S, Walter K, Kundu K, Ouwehand WH, Roberts D, Angelantonio ED, Soranzo N, Danesh J, Wheeler E, Zeggini E. Butterworth AS, Barroso I. (2020). The influence of rare variants in circulating metabolic biomarkers. PLoS Genetics, 16(3). 10.1371/journal.pgen.1008605 PMC7108731 32150548

[100] Zhu L., Liao R., Huang J., Yan H., Xiao C., Yang Y., Wang H., & Yang C. (2022). The miR-216/miR-217 Cluster Regulates Lipid Metabolism in Laying Hens With Fatty Liver Syndrome via PPAR/SREBP Signaling Pathway. Frontiers in Veterinary Science, 9. 10.3389/fvets.2022.913841 PMC9195098 35711801

[101] Kowalczyk M, Aebisher D, Szpara J, Czech S, Bartusik-Aebisher D, Henrykowska G. Molecular Mechanisms of Emerging Antidepressant Strategies: From Ketamine to Neuromodulation. Int J Mol Sci. 2025 Dec 28;27(1):344. 41516220 10.3390/ijms27010344 PMC12785913

[102] Zhao Z, Xie M, Tao S, Lv Q, Zhang J, Cai J, Liu Y, Huang Y, Liu S, Wu Y, Wang Q. Metabolic syndrome increases the risk of suicide attempt: evidence from a population-based cohort and genomic analysis. Transl Psychiatry. 2025 Oct 6;15(1):365. 41052968 10.1038/s41398-025-03575-1 PMC12501300

[103] William N, Reissner C, Sargent R, Darlington TM, DiBlasi E, Li QS, Keeshin B, Callor WB, Ferris E, Jerominski L, Smith KR, Christensen ED, Gray DM, Camp NJ, Missler M, Williams ME, Coon H. Neurexin 1 variants as risk factors for suicide death. Mol Psychiatry. 2021 Dec;26(12):7436–7445. 34168285 10.1038/s41380-021-01190-2 PMC8709873

